# Oral Immunization with *Escherichia coli Nissle 1917* Expressing SARS-CoV-2 Spike Protein Induces Mucosal and Systemic Antibody Responses in Mice

**DOI:** 10.3390/biom13030569

**Published:** 2023-03-21

**Authors:** Giovanni Sarnelli, Alessandro Del Re, Marcella Pesce, Jie Lu, Giovanni Esposito, Walter Sanseverino, Chiara Corpetti, Silvia Basili Franzin, Luisa Seguella, Irene Palenca, Sara Rurgo, Fatima Domenica Elisa De Palma, Aurora Zilli, Giuseppe Esposito

**Affiliations:** 1 Department of Clinical Medicine and Surgery, Section of Gastroenterology, University Federico II, 80138 Naples, Italy; 2 Nextbiomics S.R.L. (Società a Responsabilità Limitata), 80100 Naples, Italy; 3 Department of Physiology and Pharmacology “V. Erspamer”, Sapienza University of Rome, 00185 Rome, Italy; 4 Department of Anatomy and Cell Biology, China Medical University, Shenyang 110122, China; 5 Department of Molecular Medicine and Medical Biotechnologies, Centro Ingegneria Genetica-Biotecnologie Avanzate s.c.a rl, 80131 Naples, Italy

**Keywords:** COVID-19, engineered probiotics, oral vaccine, IgA

## Abstract

As of October 2022, the COVID-19 pandemic continues to pose a major public health conundrum, with increased rates of symptomatic infections in vaccinated individuals. An ideal vaccine candidate for the prevention of outbreaks should be rapidly scalable, easy to administer, and able to elicit a potent mucosal immunity. Towards this aim, we proposed an engineered *Escherichia coli* (*E. coli*) *Nissle 1917* (EcN) strain with SARS-CoV-2 spike protein (SP)-coding plasmid, which was able to expose SP on its cellular surface by a hybridization with the adhesin involved in diffuse adherence 1 (AIDA1). In this study, we presented the effectiveness of a 16-week intragastrically administered, engineered EcN in producing specific systemic and mucosal immunoglobulins against SARS-CoV-2 SP in mice. We observed a time-dependent increase in anti-SARS-CoV-2 SP IgG antibodies in the sera at week 4, with a titre that more than doubled by week 12 and a stable circulating titre by week 16 (+309% and +325% vs. control; both *p* < 0.001). A parallel rise in mucosal IgA antibody titre in stools, measured via intestinal and bronchoalveolar lavage fluids of the treated mice, reached a plateau by week 12 and until the end of the immunization protocol (+300, +47, and +150%, at week 16; all *p* < 0.001 vs. controls). If confirmed in animal models of infection, our data indicated that the engineered EcN may be a potential candidate as an oral vaccine against COVID-19. It is safe, inexpensive, and, most importantly, able to stimulate the production of both systemic and mucosal anti-SARS-CoV-2 spike-protein antibodies.

## 1. Introduction

Several COVID-19 vaccines are now available, and mass vaccination campaigns in first-world countries have consistently abated the rates of symptomatic disease, especially severe disease [1]. Nonetheless, on the one hand, vaccinees offer protection against severe disease; on the other hand, immunization campaigns have appeared to have a less significant impact on the infection chain of control, which is equally as important from a public health standpoint [2]. Infections and disease spreading among vaccinated individuals several months post-injection is now increasingly common, especially with recently emerging variants [3]. This should be expected since intramuscularly injected vaccines are more likely to induce a robust systemic immune response to protect against disease progression, rather than a mucosal immune response that might also prevent the viral entry at oral and nasal cavities [4]. Furthermore, protection rates against symptomatic infection could drop to 80% due to waning immunity, indicating that repeated booster immunizations could be required to prevent breakthrough infections in vaccinated individuals [5]. This poses the question: since the currently available vaccines are costly; require specialized personnel and sterile equipment for administration; and need a strict cold supply chain to be transported and maintained, will they be able to assure mass coverage beyond first-world countries? Moving forward, developing innovative vaccine platforms that are able to produce economical, rapidly scalable vaccines with simpler storage and delivery could offer a potential solution [6]. Engineered probiotics have facilitated the release of therapeutically active molecules in the intestines [7]. More interestingly, engineered probiotic strains offer the prospect of expressing foreign antigens on their surfaces in order to facilitate the interaction between specific antigens and the mucosal immune system of the gastrointestinal tract, with the obvious advantages of eliciting a potent mucosal immune response [8]. In addition, other authors have previously hypothesized the application of non-pathogenic probiotic species as a safe and feasible platform to obtain a readily manufacturable and inexpensive oral COVID-19 vaccine [9]. A number of bacteria have been investigated as delivery systems to express recombinant proteins. For example, E. coli represents one of the most studied and employed microorganisms due to its safety profile, high growth rate, genomic simplicity, and ease of handling [10,11,12]. A proof-of-concept study introduced a killed whole-genome-reduced E. coli vaccine with a surface expression of the SARS-CoV-2 fusion peptide, and the authors reported the feasibility of this platform against SARS-CoV-2. Although promising, this approach failed to elicit strong neutralizing humoral immune responses against the fusion peptides in a porcine model [13].

In our model, we genetically engineered the apathogenic EcN strain in order to express the SARS-CoV-2 spike protein (SP) on the bacterial surface, given the high immunogenicity of the bacteria [14]. Towards this aim, we engineered an EcN strain with an SP-coding plasmid (EcN-pAIDA1-SP) using the adhesin involved in diffuse adherence 1 (AIDA1) as an autotransporter, in order to enable the surface expression of SARS-CoV-2 SP on this otherwise apathogenic bacteria. The surface expression of the viral epitope by commensal non-pathogenic bacteria could prolong the half-life of the epitope once administered to an immunized animal, while also boosting the immune response due to the surface proteins of gram-negative bacteria acting as adjuvants [15,16]. The engineered *E. coli* was then orally administered in mice to assess its in vivo efficacy in stimulating the gut-associated lymphoid tissue (GALT) in order to produce systemic anti-SARS-CoV-2 spike IgG and secretory anti-SARS-CoV-2 spike IgA at the mucosal level, both in the gut and lung. Then, the neutralization capacity of the specific anti-SARS-CoV-2 spike protein antibodies were assessed.

## 2. Materials and Methods

### 2.1. Generation of E. coli Nissle Expressing SARS-CoV-2 Spike Protein on Cell Membrane as Immunogen

The adhesin involved in diffuse adherence (AIDA) of *E. coli* has been used for the expression of recombinant proteins on the outer membrane of E. coli. The surface translocation system of AIDA-I is an anchor protein from the *Escherichia coli (Escherichia coli strain 2787*). Surface expression using AIDA required three parts: signal peptide, passenger domain, and anchor protein AIDAc. The signal peptide aided in transporting the protein to the membrane, the passenger domain was the protein of interest, and the AIDAc was the anchor protein, whose β-barrel structure could be anchored on the outer membrane, so that the protein of interest could be expressed on the surface. A specific plasmid pAIDA1 was exploited to express the SARS-CoV-2 SP on the EcN membrane.

The plasmid coding for the SARS-CoV-2 SP was purchased from Genewiz, Suzhou, China, product cat n° GS-200519_A001 (https://climsprod.genewiz.com.cn/ECProduct/Products/S-in-pCDNA3-1, accessed on 3 February 2022), and we cloned the sp ike from this plasmid for the pAIDA1 vector. The primers for RTPCR are listed below (the red sequences are flanking sequences around the KpnI/SacI sites from pAIDA1 vector (Addgene 79180, http://www.addgene.org/79180/, accessed on 3 February 2022), which were used for overlapping in-fusion in the next step:

Spike-FW2: CAGGGTCCGGGTACCATGTTTGTTTTTCTTGTTTTATTGC

Spike-RV2: CAGGTTTTCGAGCTCTGTGTAATGTAATTTGACTCC

The pAIDA1 vector was digested with KpnI/SacI, and the PCR product was inserted into the cutting site by using the in-fusion method from Clontech, resulting in the pAIDA1-SP. The plasmid was transduced into the EcN and confirmed by Western blotting and immunofluorescent microscopy for the expression of the spike protein on the outer membrane of the EcN.

### 2.2. Immunofluorescent Analysis of Spike Protein Expression on the Bacterial Surface

According to the procedure previously described [17] with slight modifications, immunofluorescence analysis was performed in both EcN-pAIDA1-SP and in the “empty-plasmid” transfected EcN-pAIDA1 as a negative control. Briefly, bacteria were isolated in petri dishes with LB Agar supplemented with chloramphenicol (25 μg/mL) and incubated at 37 °C overnight. Then, a single colony was cultured in LB broth (Difco) for 24 h at 37 °C in presence of isopropyl β-d-1-thiogalactopyranoside (IPTG) 1 mM and then centrifuged at 3600 RPM for 15 min at 37 °C; the pellets were washed in phosphate buffer saline (PBS). For immunofluorescence staining of both the EcN-pAIDA1 and the EcN-pAIDA1-SP, 1 × 10^7^ bacterial cells were placed on polyethyleneimine-coated coverslips and fixed with 4% PFA. Blocking solution containing 1% bovine serum albumin (BSA) in PBS (*w*/*v*) was used. Labelling was performed using polyclonal rabbit anti-SARS-CoV-2 spike protein S1 antibody (1:100 dil. *v*/*v*) (Cell Signaling Technology, Inc., Danvers, MA, USA). Secondary fluorescein isothiocyanate-conjugated anti-rabbit antibodies were incubated at room temperature for 2 h in the dark. Samples were examined by Optika XDS-3L4 microscope (Ponteranica, Bergamo, Italy). Images were captured at 100× by a high-resolution digital camera (Nikon Digital Sight DS-U1).

### 2.3. Western Blot Analysis of Spike Protein Expression

Membrane protein fractions were extracted from EcN-pAIDA1-SP and EcN-pAIDA1 pellets and processed by Western blot analysis, as previously described by Jarmander et al. [18] with slight modifications. Stool samples collected at week 16 were also processed by Western blot analysis. Briefly, protein samples were diluted 3:1 in 4× SDS loading buffer (Fermenta-Thermo Fisher Scientific, Waltham, MA, USA), incubated at 105  °C for 15 min, and then loaded onto 10% NuPage Bis-Tris gels (Invitrogen-Thermo Fisher Scientific, MA, USA) for electrophoresis. The protein concentration was determined using the Bradford assay. After the transfer, the membranes were blocked overnight at 4 °C in PBS containing 5% *w*/*v* non-fat milk powder (Sigma-Aldrich Corporation-Merk KGaA, St. Louis, MO, USA) and then incubated with rabbit SARS-CoV-2 spike protein S1 polyclonal antibody (1:1000 dil. *v*/*v*) (Invitrogen, Waltham, MA, USA). Membranes were then incubated with the specific secondary antibodies conjugated to HRP. Immune complexes were exposed to enhanced chemiluminescence detection reagents, and the blots were analyzed by scanning densitometry (Versadoc MP4000; Bio-Rad, Segrate, Italy). Results were expressed as optical density (OD; arbitrary units = mm^2^).

### 2.4. Animals, Immunization Protocol, and Sample Collection

**Scheme 1:**
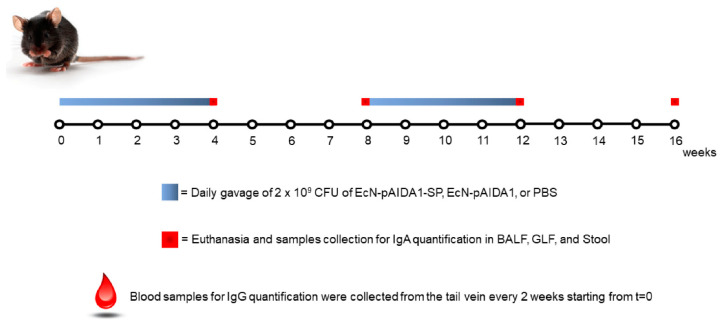
Immunization protocol and sample collection during the 16 weeks.

All experiments involving animals were carried out according to the Sapienza University’s Ethics Committee (Organizzazione per il benessere animale, OPBA), approval code 890/2021-PR, which was approved on 17 November 2021. All animal experiments complied with the ARRIVE guidelines and were carried out in accordance with the U.K. Animals (Scientific Procedures) Act, 1986, and associated guidelines, E.U. Directive 2010/63/EU for animal experiments. Eight-week-old male C57BL/6 mice were used for the experiments (Charles River, Lecco, Italy). All mice were maintained on a 12 h light/dark cycle in a temperature-controlled environment with access to food and water *ad libitum*. The mice were sacrificed at the various time-points (see Scheme 1) by CO_2_ hypoxia. Mice were randomly divided into 3 groups (control, EcN-pAIDA1 and EcN-pAIDA1-SP) that comprised 16 animals each. The entire protocol lasted 16 weeks, and the 3 groups received, depending upon the experimental protocol, (1) intragastric gavage of phosphate buffer saline (PBS); (2) intragastric gavage of a suspension of the EcN-pAIDA1 as a negative control; and (3) intragastric gavage of the EcN-pAIDA1-SP suspension. For the oral route, 2 × 10^9^ CFU of the EcN-pAIDA1-SP and EcN-pAIDA1 in a volume of 150 μL of sterile PBS were administered daily via intragastric gavage from weeks 0 to 4 and from weeks 8 to 12. A total of N = 4 animals were sacrificed at weeks 4, 8, 12, and 16. Blood samples were collected from the tail vein [19] on weeks 0 (pre-immunization), 2, 4, 6, 8, 10, 12, 14, and 16. Blood samples were stored at 37 °C for 1 h, and then the serum was separated from blood cells by centrifuging at 12,000 RPM for 5 min [20]. The sera samples were stored at −80 °C until they were analyzed. After sacrificing the animals, the bronchoalveolar (BALF) and gut (GLF) lavage fluids were obtained by washing the respective organs 3 times with 1 mL of ice-cold PBS containing protease inhibitors 10 µL/mL (Sigma-Aldrich, St. Louis, MO, USA) at weeks 4, 8, 12, and 16. Lavage fluids were centrifuged at 2500× *g* for 20 min at 4 °C, and the supernatants were stored at −20 °C until their analysis [21]. IgA extraction was performed, as previously described by Smeekens et al. [22], with slight modifications. Briefly, stool samples were collected at weeks 0, 4, 8, 12, and 16 and kept on ice. Rapidly sterile phosphate-buffered saline (PBS) (supplemented with 10% goat serum (Fisher Scientific, Waltham, MA, USA) 0.05% NaN_3_ and 10 µL/mL protease inhibitor (Sigma-Aldrich, St. Louis, MO, USA)) was placed in a tube per 10 mL/mg of stool and then vortexed for 20 min to disrupt the pellets. After centrifuging at 13,000 RPM for 10 min at 4 °C in the microcentrifuge tube, the supernatant collected was stored at −20 °C until analysis.

### 2.5. Assessment of Safety Profile and Adverse Effects

The disease activity index (DAI) score was used to exclude gastrointestinal side effects. The DAI index was calculated as the total score (body weight decrease + stool consistency + rectal bleeding) divided by 3, as previously reported by Cooper et al. [23]. Bodyweight, stool consistency, and the presence of gross blood in stools were evaluated daily and scored for each mouse during the experimental period. Rectal temperature was measured daily for the entire duration of the experiment. To obtain the rectal temperature, the mice were hand-restrained and placed on a horizontal surface. The tail was then lifted, and the probe (covered with Vaseline) was gently inserted into the rectum, up to a fixed depth [24].

### 2.6. ELISA for Specific Anti-SARS-CoV-2 S Antibodies Detection

Specific anti-SARS-CoV-2-S protein IgG quantification was performed on the sera samples collected every 2 weeks (0, 2, 4, 6, 8, 10, 12, 14, and 16), using Mouse Anti-2019 nCoV(S)IgG ELISA Kit 96T. Specific anti-SARS-CoV-2-S protein IgA levels in stools as well as the BALF and GLF samples were assessed every 4 weeks (0, 4, 8, 12, and 16) using Mouse Anti-2019 nCoV(S)IgA ELISA Kit 96T. All the ELISA analyses were performed according to the manufacturer’s instructions (Fine Biotech Co., Wuhan, China).

### 2.7. Relative Avidity Index for Anti-SARS-CoV-2 SP IgA and IgG

Plasma, GLF, and BALF samples were tested for antibody avidity by a Mouse Anti-2019 nCoV(S)IgA and Anti-2019 nCoV(S)IgG ELISA kit equipped with recombinant SARS-CoV-2 2019 spike protein pre-coated wells (Fine Biotech Co., Wuhan, China). To determine the relative avidity index (RAI), two microplate wells were used for each analysis. In one well, the anti-SARS-CoV-2 ELISA was carried out according to the manufacturer’s instructions while an additional urea treatment (5.5 M for 10 min) was performed in the other well in order to detach the low-avidity antibodies from the antigen [25,26,27]. The RAI was calculated as the ratio of the absorbance with and without urea incubation and expressed as a percentage and fold increase vs. control group.

### 2.8. Immunofluorescence Analysis

On week 16, the animals were sacrificed, and the colon and spleen were isolated, fixed in ice-cold 4% paraformaldehyde (PFA), and sectioned into 20 μm slices. Colon sections were blocked with bovine serum albumin and subsequently stained with mouse anti-CD103 antibody (1:100 dilution *v*/*v*; Proteintech, Manchester, UK) or mouse anti-CD138 antibody (1:100 dilution *v*/*v*; Novus Biologicals, Abingdon, UK). Spleen sections were similarly processed and stained with rabbit anti-CD138 antibody (1:100 dilution *v*/*v*; Novus Biologicals, Abingdon, UK). Slices were then washed with PBS 1X and incubated in the dark with fluorescein isothiocyanate-conjugated appropriate secondary antibodies (Abcam, Cambridge, UK). Nuclei were stained with Hoechst. Sections were analyzed by microscope (Nikon Eclipse 80i), and images were captured by a high-resolution digital camera (Nikon Digital Sight DS-U1).

### 2.9. Histopathological Analysis

After sacrifice, mouse distal colons were fixed in 4% paraformaldehyde (PFA), sectioned into 15 μm slices, and stained with hematoxylin and eosin (H&E) for macroscopic and histopathological assessment. Colonic histological damage was evaluated through a combined score, according to the criteria proposed by Li et al. and considering the following parameters: (i) distortion and loss of crypt architecture (0 = none; 1 = mild; 2 = moderate; 3 = severe); (ii) infiltration of inflammatory cells (0 = normal; 1 = mild infiltration; 2 = moderate infiltration; 3 = dense infiltration); (iii) muscle thickening (0 = normal; 1 = mild muscle thickening; 2 = moderate muscle thickening; 3 = marked muscle thickening); (iv) goblet cell depletion (0 = absence; 1 = presence); and (v) crypt absence (0 = absence; 1 = presence). Slices were analyzed by microscope, Nikon Eclipse 80i (Nikon Corporation, Tokyo, Japan), and the images were captured at 4× magnification by a high-resolution digital camera (Nikon Digital Sight DS-U1). Cumulative histological damage scores were expressed as average scores in each experimental group.

### 2.10. Enzyme-Linked Immunosorbent Assay for LPS

Enzyme-linked immunosorbent assay (ELISA) for LPS (Thermo Fisher Scientific, MA, USA) was carried out on mouse plasma according to the manufacturer’s protocol. Absorbance was measured on a microtiter plate reader. LPS levels were determined using standard curve methods.

### 2.11. Statistical Analysis

Results are expressed as the mean ± standard deviation (SD) of n experiments performed in triplicate, depending upon the experiment (see figure legends). Statistical analyses were performed using one-way ANOVA, and multiple comparisons were performed using a Bonferroni post hoc test. * *p* < 0.05, ** *p* < 0.01, and *** *p* < 0.001 indicated a statistically significant difference vs. control group.

## 3. Results

### 3.1. Expression of SARS-CoV-2 Spike Protein by Engineered E. coli Nissle

**Figure 1:**
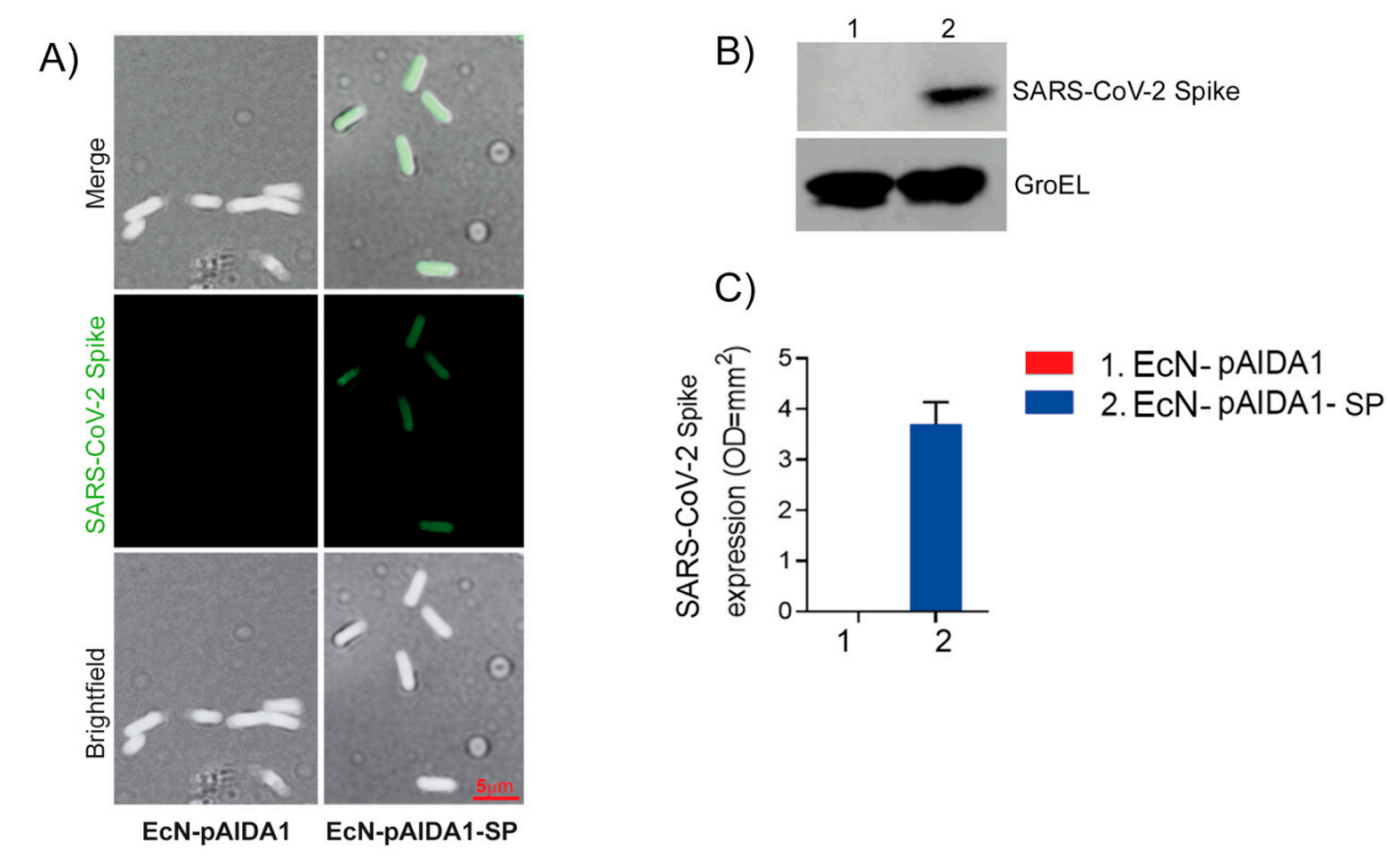
EcN-pAIDA1-SP expresses SARS-CoV-1 spike protein on the cell surface as shown by immunofluorescence and brightfield (100× magnification) (**A**) and Western blot analysis for SARS-CoV-2 spike protein S1 quantification (**B**,**C**), (see Supplementary Figure for uncropped blot). Results are expressed as mean ± SD of n = 4 experiments performed in triplicate.

The presence of the AIDA1-SP complex was evaluated by immunofluorescence on the bacterial surface (Figure 1A) and by Western blot analysis on the bacterial membrane lysate. Immunohistochemistry revealed the selective expression of the SARS-CoV-2 spike protein on the bacterial surface of the EcN-pAIDA1-SP, while the SARS-CoV-2 spike protein was not detected on the EcN-pAIDA1 bacterial cells, which were used as negative control (Figure 1A). The localization of the SARS-CoV-2 spike protein on the surface of the EcN-pAIDA1-SP was further verified by a Western blot analysis of the membrane fraction of the bacteria lysates. Our analysis revealed the presence of the SARS-CoV-2 spike protein’s immunoreactive bands in samples of the EcN-pAIDA1-SP membrane fraction lysate, but not in the EcN-pAIDA1 (Figure 1B,C).

### 3.2. EcN-pAIDA1-SP Is Capable of Expressing and Delivering the SARS-CoV-2 SP to the Intestinal Site

**Figure 2:**
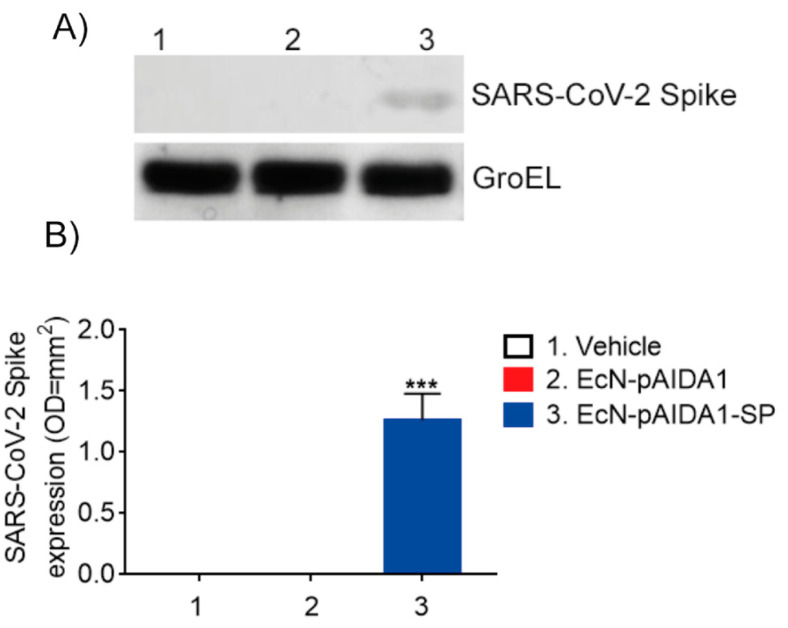
Expression of SARS-CoV-1 spike protein in fecal samples of the EcN-pAIDA1-SP, EcN-pAIDA1, and control mice. Western blot analysis for SARS-CoV-2 spike protein S1 quantification (**A**,**B**). Results are expressed in mean ± SD of n = 4 experiments performed in triplicate. (*** *p* < 0.001 vs. control group). (See Supplementary Figure for uncropped blots).

The presence of the SP at the intestinal site was evaluated by the Western blot analysis that had been performed on the fecal samples collected at week 16. The Western blot analysis revealed the presence of the SARS-CoV-2 PS in the stool of the EcN-pAIDA1-SP-treated animals, while the SP was not detected in the samples of the EcN-pAIDA1 and control groups, respectively (Figure 2A,B). These results indicated the ability of orally administered EcN-pAIDA1-SP to deliver SARS-CoV-2 SP amid the intestinal milieu.

### 3.3. Engineered EcN-pAIDA1-SP Elicits a Time-Dependent Increase in Circulating Anti-SARS-CoV-2 SP IgGs

We performed a 16-week immunization protocol that entailed the daily intragastric administration of the EcN-pAIDA1-SP for 4 consecutive weeks (weeks 0–4) with a subsequent immunization booster for 4 weeks (weeks 8–12). The sera samples, collected at 2-week intervals, were analyzed by ELISA assay to determine the total amount of specific IgG.

**Figure 3:**
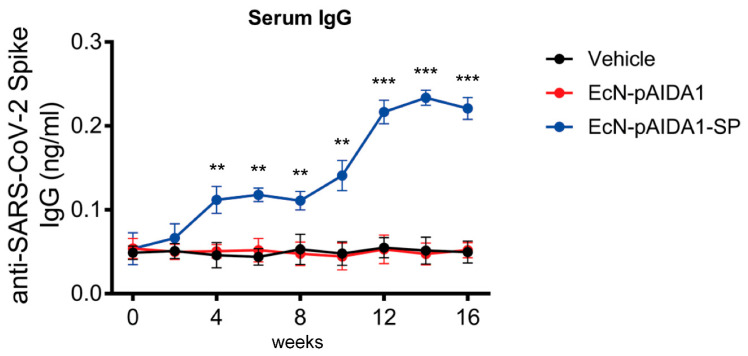
*E. coli* Nissle pAIDA1-Spike triggered IgG(s) production after oral administration. IgG titre in sera samples of mice treated with control (black), EcN-pAIDA1 2 × 10^9^ CFU (red), and EcN-pAIDA1-SP 2 × 10^9^ CFU (blue) at weeks 0, 2, 4, 6, 8, 10, 12, 14, and 16. Results are expressed in mean ± SD of n = 4 experiments performed in triplicate. (** *p* < 0.01 vs. control group, *** *p* < 0.001 vs. control group).

During the first 2 weeks (weeks 0–2), the levels of detected anti-SARS-CoV-2-SP IgG antibodies were comparable among the 3 groups, along with very low levels of circulating immunoglobulins in the immunized mice. However, starting from the fourth week, a significant increase in the IgG titre (+121% vs. control; *p* < 0.01) was detected in the EcN-pAIDA1-SP group, as compared to the empty vector. The antibody titre remained stable over time for the following 4 weeks before the subsequent immunization booster (+127% and +118% vs. control, at week 6 and 8, respectively; both *p* < 0.01). Following the booster at week 8, we observed a steady increase in the anti-SARS-CoV-2 SP IgG titres, starting at week 10 (+200% vs. control; *p* < 0.01), and the levels of circulating IgG antibodies more-than-doubled by week 12 (+309% vs. control; *p* < 0.001). At the end of our timeline (weeks 14 and 16), the anti-SARS-CoV-2 SP IgG levels in the EcN-pAIDA1-SP group had slightly increased, as compared to the last time point (week 14, +334% vs. control; *p* < 0.001), remaining consistently at this level until the end of the experiment (week 16, +325% vs. control; *p* < 0.001) (Figure 3). Conversely, no IgG levels above this level were detected after the short-term immunization protocol (Supplementary Figure S1A).

### 3.4. Engineered EcN-pAIDA1-SP Induces a Specific IgA-Mediated Mucosal Immune Response at Gastrointestinal and Pulmonary Interfaces

To assess the specific mucosal immune responses, we evaluated the total IgA levels of the anti-SARS-CoV-2-SP in the gut lavage fluids (GLF), the bronchoalveolar lavage fluids (BALF), and the stool samples.

**Figure 4:**
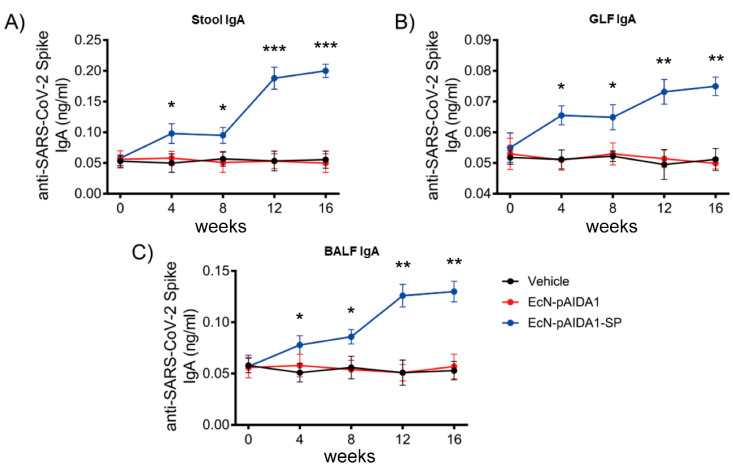
E. coli Nissle pAIDA1-Spike triggered IgA(s) production after oral administration. IgA titre in samples of mice treated with control (black), EcN-pAIDA1 2 × 10^9^ CFU (red), and EcN-pAIDA1-SP 2 × 10^9^ CFU (blue) at weeks 0, 4, 8, 12, and 16. (**A**) IgA titre in fecal samples of mice treated with gavage of PBS (black), EcN-pAIDA1 d 2 × 10^9^ CFU (red), and EcN-pAIDA1-SP 2 × 10^9^ CFU (blue). (**B**) IgA titre in GLF samples of mice treated with gavage of PBS (black), EcN-pAIDA1 d 2 × 10^9^ CFU (red), and EcN-pAIDA1-SP 2 × 10^9^ CFU (blue). (**C**) IgA titre in BALF samples of mice treated with gavage of PBS (black), EcN-pAIDA1 d 2 × 10^9^ CFU (red), and EcN-pAIDA1-SP 2 × 10^9^ CFU (blue). Results are expressed in mean ± SD of n = 8 experiments performed in triplicate. (* *p* < 0.05 vs. control group, ** *p* < 0.01 vs. control group, *** *p* < 0.001 vs. control group).

The administration of the EcN-pAIDA1-SP induced a significant increase in the anti-SARS-CoV-2-spike IgA titre in stool samples. Furthermore, at week 4, the fecal IgA levels of the EcN-pAIDA1-SP group were significantly higher, as compared to the control group (+69% vs. control; *p* < 0.05), and they remained steadily increased at week 8 (+64% vs. control; *p* < 0.05). A significant increase in the fecal IgAs in the EcN-pAIDA1-SP group was observed at the end of the immunization booster (week 12, +123% vs. control; *p* < 0.001), with a titre that continued to increase until week 16 (+300% vs. control; *p* < 0.001) (Figure 4A). Conversely, no IgA levels above the previous levels were detected following the short-term immunization protocol (Supplementary Figure S1B). The results observed in the stools were also replicated by assessing the IgA levels of the anti-SARS-CoV-2 SP in the GLFs of the treated mice, which steadily increased at week 4 (+28.4% vs. control; *p* < 0.05) and then remained stable until week 8 (+ 27.2% vs. control; *p* < 0.05). Following the booster, at week 12, the IgA levels continued to progressively increase, similar to the increase that had been observed in the circulating IgG titre. Indeed, we observed a nearly 2-fold increase in the titre of the mucosal IgA in the GLFs of the EcN-pAIDA1-SP group at the end of the immunization booster (week 12, +43.5% vs. control; *p* < 0.01), and this continued up until the end of the experiment, at week 16 (+47% vs. group 2; *p* < 0.01) (Figure 4B). Interestingly, the increase in the anti-SARS-CoV-2 SP IgA observed in the BALF of the EcN-pAIDA1-SP group was even higher than the levels observed in the GLF. In particular, at the end of the fourth week, the levels of the BALF anti-SARS-CoV-2 SP IgA were doubled at week 4, as compared to the control group (week 4, +50% vs. control; *p* < 0.05) and continued to slightly increase until week 8 (+65.4% vs. control; *p* < 0.05). By the end of week 12, the levels of the anti-SARS-CoV-2 SP IgA of the EcN-pAIDA1-SP group increased dramatically, remaining constant until the end of the experiment (+142.3% and +150% vs. control at weeks 12 and 16; both *p* < 0.01) (Figure 4C). In contrast, only the background levels of antibodies were detected in the control and the EcN-pAIDA1 groups.

### 3.5. Engineered EcN-pAIDA1-SP Increases the Dendritic Cell Activity, Increases Plasma Cell Numbers in Both Colon and Spleen

**Figure 5:**
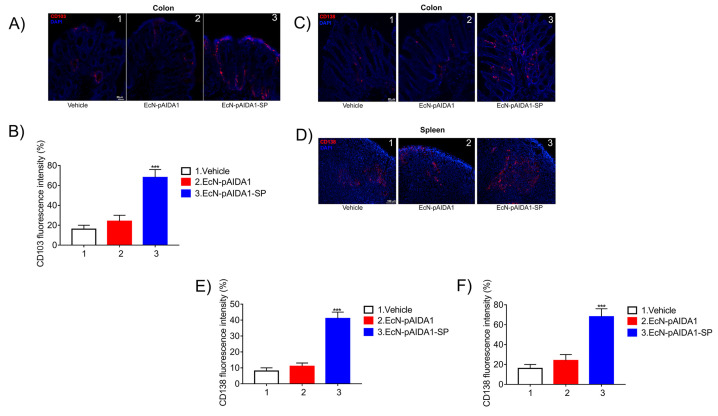
Immunofluorescence staining and their respective quantification corresponding to (**A**,**B**) CD103 and (**C**–**F**) CD138 at the colon and spleen sites, showing the effects of the EcN-pAIDA1-SP on mucosal dendritic cells in the gut (**A**) and on plasma cell population in the colonic mucosa and at the spleen site. All the samples of EcN-pAISA1-SP immunized group, EcN-pAIDA1 group, and control group were collected at week 16. Nuclei were stained using Hoechst staining. Results are expressed as mean ± SD of n = 5 experiments performed in triplicate. *** *p* < 0.001 vs. control. Scale bar = 20 μm (**A**,**C**); 100 μm (**D**).

In order to investigate the ability of the EcN-pAIDA1-SP to stimulate an immune response, we performed immunofluorescence analysis at the gut and spleen sites after 16 weeks of immunization. Our results showed a significantly higher expression of CD103 immunoreactivity, as a marker of dendritic cells, in the gut mucosa of the EcN-pAIDA1-SP, as compared to the EcN-pAIDA1-treated and control mice (+180% vs. control, +138 vs. EcN-pAIDA1; *p* < 0.01) (Figure 5A,B). In the colonic mucosa of the EcN-pAIDA1-SP group, an increased expression of the plasma cell marker CD138 was also observed (+325% vs. control, +183% vs. EcN-pAIDA1; *p* < 0.001) (Figure 5C). Similar to the colon, a significantly increased expression of CD138 immunoreactivity was observed in the spleen in the EcN-pAIDA1-SP-treated group (+412% vs. control, +272% vs. EcN-pAIDA1; *p* < 0.001) (Figure 5D). Overall, these results demonstrated that EcN-pAIDA1-SP was able to activate both the mucosal and systemic immunity.

### 3.6. Anti-SARS-CoV-2 SP IgG and IgA Exhibit a High Relative Avidity Index (RAI) for SP

**Figure 6:**
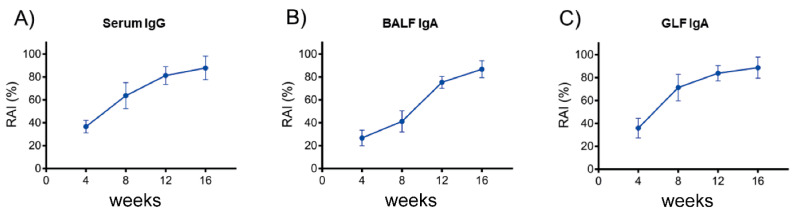
Specific anti-SARS-CoV-2 spike IgG antibody avidity detected in sera samples of EcN-pAISA1-SP immunized group at weeks 4, 8, 12, and 16 (**A**). Specific anti-SARS-CoV-2 spike IgA antibody avidity detected in BALFs samples of EcN-pAIDA1-SP immunized group at weeks 4, 8, 12, and 16 (**B**). Specific anti-SARS-CoV-2 spike IgA antibody avidity detected in GLFs samples of EcN-pAISA1-SP immunized group at weeks 4, 8, 12, and 16 (**C**). Results were calculated as ODurea/ODreference and express as a percentage of n = 4 experiments performed in triplicate.

When testing the avidity of the serum anti-SARS-CoV-2 SP IgG produced in the mice after EcN-pAIDA1-SP immunization at 4, 8, 12, and 16 weeks, we observed a noticeable trend of strongly bound SP, which had been used to coat the wells. All the tested samples displayed increased RAI values at the evaluated time-points. Our results indicated an average RAI of approximately 90% for IgG in the tested sera samples of the EcN-pAIDA1-SP group at week 16 (Figure 6A). Similarly, the BALF-derived IgAs from the EcN-pAIDA1-SP group demonstrated an average RAI of around 80% at week 16 (Figure 6B). Similar results were obtained from the GLF samples, with the IgA produced in the gut of the EcN-pAIDA1-SP group showing an average RAI that was higher than 80% at week 16 (Figure 6C).

### 3.7. EcN-pAIDA1-SP Immunization Did Not Induce Side Effects or Affect Mice Survival

**Figure 7:**
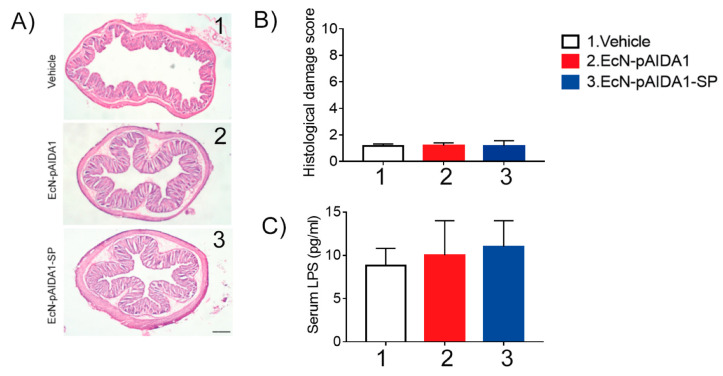
Representative images of hematoxylin-and-eosin (H&E) stained colon sections (**A**) and relative histological damage score showing the effect of the EcN-pAIDA1-SP (**B**); magnification 4×; samples collected at t = 16 weeks. Serum levels of LPS after oral administration of control, EcN-pAIDA1, and EcN-pAIDA1-SP; samples collected at *t* = 16 weeks (**C**). Results are expressed as mean ± SD of n = 4 experiments performed in triplicate.

No significant changes in the disease activity index (DAI) score in the EcN-pAIDA1-SP immunized mice were observed, as compared to the EcN-pAIDA1 and the control groups. No significant variations were observed between the three groups in terms of body weight and stool consistency, and none of the animals ever received a score higher than one in any of the analyzed parameters. Moreover, rectal bleeding was not observed in the groups. Body temperature was also unaffected. This parameter was not altered by the EcN-pAIDA1-SP immunization, and the body temperature in the immunized group ranged from 36.1 °C to 36.8 °C (see Supplementary Figure S2A,B). Similarly, no differences were observed in the mucosal integrity among the 3 groups (Figure 7A,B), and the serum levels of the LPS also showed no significant differences (Figure 7C). Finally, no differences in the survival of the EcN-pAIDA1-SP immunized mice were observed in comparison with the other two groups during the entire time course of our experiment, confirming the safety of the EcN-pAIDA1-SP under these conditions.

## 4. Discussion

As SARS-CoV-2 infections continue to rise even among vaccinated individuals [28,29], there is an urgent call for an alternative vaccine platform that is less expensive, readily scalable, as well as easier to store and administer, as this would overcome the major limitations of the current COVID-19 vaccines [30]. Other authors have previously suggested [31,32] the use of probiotic strains as delivery systems for viral or bacterial antigens with promising results, including SARS-CoV-2 [13,33]. In this study, we proposed a new immunization approach using plasmid transfection by inducing the widely used apathogenic EcN strain to express and expose SARS-CoV-2 SP on the bacterial surface.

One advantage of using orally administered vaccines includes simpler logistics, as they do not require a cold-supply chain or cold storage, nor do they need specialized personnel or sterile syringes to administer the vaccine. This would realistically meet the needs in remote regions of the world and in economically disadvantaged nations. Though less apparent, the oral route has another crucial advantage over intramuscular-injected vaccines, as the gastrointestinal tract plays a primary role in the host immunity to pathogens, including viruses, by eliciting a potent mucosal immune response with the production of the secretory IgA [34]. The GALT is responsible for at least 70% of the potential antibody response in mammals [35], and IgAs are antibodies on the front lines of viral infections, and they support the mucosal immunity that is crucial to prevent virus colonization in the host cells [36].

Remarkably, a recent report exploring the effects of the mRNA BNT162b2 vaccine on both total IgG and IgA antibodies in the saliva and serum indicated that intramuscular administration of the vaccine induced a strong systemic immune response, with high levels of serum IgG, with low titres of salivary IgG and even lower IgA in immunized individuals. The authors speculated that this effect could be related to a less effective mucosal immune response, as compared to other immunization routes (nasal or oral) and suggested that we should “reconsider the strategy of vaccination to prevent not only the severe disease but the viral infection (i.e., the so-called ‘sterilizing immunity’)” [4].

To the best of our knowledge, this was the first study exploring the preclinical effects of a COVID-19 oral vaccine on both mucosal and systemic immunity.

Our findings demonstrated that starting at week 4 and using an oral administration of the EcN-pAIDA1-SP, significant titres of specific anti-SARS-CoV-2 SP IgA were detectable in both the lungs and intestinal secretions, as well as in the stool of treated mice. These results suggested that the EcN-pAIDA1-SP was capable of colonizing the intestinal lumen, expressing the functional construct AIDA1-SP, and triggering mucosal and systemic Ig(s)-mediated immune responses in the mucosa-associated lymphoid tissues. The presence of high levels of the specific IgA anti-SARS-CoV-2 SP in the BALF was of paramount importance, considering the prominent role of the respiratory tract as a first-line defense against SARS-CoV-2 transmission and could be crucial for preventing the viral colonization of the mucosa [37].

By using the oral administration of the EcN-pAIDA1-SP, systemic immunity was triggered, as anti-SARS-CoV-2 spike IgG were detectable in serum samples two weeks after the beginning of the treatment. From the fourth week onward, the circulating IgG titre was significantly higher, as compared to both control and native EcN groups. Similar to IgA, the plasmatic concentrations of IgG continued to increase over time, doubling by the end of the 16th week. Furthermore, both the serum IgG and the secretory IgA displayed a progressively increased avidity index, indicating the selective binding to the spike protein.

Though it is not a gold-standard methodology, antibody avidity could be useful in assessing vaccination efficacy [38], and our results were consistent with a progressively strengthened bind between the immunoglobulins and the epitope, as the immune response matured after the booster administration. Overall, our data demonstrated the efficacy of a bacterial-based immunization system in stimulating a specific and enduring humoral immune response against SARS-CoV-2 SP. Furthermore, our data suggested the possibility that EcN-pAIDA1-SP could prevent the invasion and colonization of the intestinal and respiratory mucosa by triggering an IgA-mediated immune response.

Our findings demonstrated the excellent preclinical efficacy of the proposed bacterial vector vaccine in a murine model. However, there were some limitations. For example, we did not test the efficacy of the EcN-pAIDA1-SP in a viral model of SARS-CoV-2 infection. Moreover, there were several limitations related to the use of a murine model, in terms of the predictability of adverse reactions and the translatability to humans. However, during our experiment, none of the animals ever showed signs of colitis-like symptoms or fever. The complete absence of adverse reactions was predictable since several veterinary vaccines have been based on bacterial vectors.

To unequivocally establish the safety of our transfected *E. coli Nissle*, further analysis is required, including translating from murine to primate/human models to better predict the potential adverse reactions. Nevertheless, we are quite confident that EcN-pAIDA1-SP will not show any adverse reactions, since the chosen antigen carrier *E. coli Nissle* is a well-known commensal bacterium that is fully integrated into human gut microbiota [39,40,41] and is able to exert immunomodulatory functions that reduce the inflammatory process. It has been demonstrated that EcN could trigger the production of anti-inflammatory mediators, such as IL-10, and suppress the immune response of lymphocyte T, macrophages, and other immunocompetent cells of both specific and nonspecific immunity, even at the systemic site [42,43,44]. In addition, EcN stimulated the systemic production of antibodies of B lymphocytes associated with the mucous membranes and induced the systemic production of antibodies (IgM, IgA) in adults [45]. The intestinal microenvironment and the engineered probiotic could, therefore, create a highly efficient and immunological-regulated antibody response on site [46].

In addition to the aforementioned advantages related to the administration route, which provided better compliance and easier self-administration, several other benefits may result from this immunization approach. For example, the proposed oral vaccine platform enables a shortened production time. Since the antigen carrier is a living organism and able to replicate exponentially in the correct environment, it has a faster production time, as compared to the other formulations available on the market. In addition, vaccine storage would be more efficient, considering that all probiotics readily endure the lyophilization process [47] and do not require particularly low storage temperatures. Moreover, our vaccine platform could easily be modified for newly emerging variants by adapting the plasmid construct in a timely and readily manufacturable manner and, therefore, could prevent surges of breakthrough infections and new pandemic waves.

Our preliminary results may pave the way for second-generation vaccines that are safer, more affordable, and rapidly scalable, and they may promote the goal of obtaining total immunity by stimulating the IgA-mediated immune response.

## 5. Patents

Engineering of probiotic e. Coli nissle 1917 expressing the SARS-CoV-2 spike protein as a chimeric model of intestinal immunization against COVID-19 (wo2022219530 (a1)―2022-10-20).

## Data Availability

The datasets generated during and/or analyzed during the current study are available from the corresponding authors upon reasonable request. The DNA sequences generated during the current study are available in the AddGene repository, [http://www.addgene.org/79180/, accessed on 8 February 2022].

## Supplementary Materials

The following supporting information can be downloaded at: https://www.mdpi.com/article/10.3390/biom13030569/s1, Scheme S1: Experimental time course for the short-term immunization; Figure S1: Anti-SARS-CoV-2 spike protein antibody titre (ng/mL) in serum IgG (A) and stool IgA (B) following a short-term immunization protocol with daily intragastric gavage at day 1, 2, 3, and 4 of the EcN-pAIDA1-SP; Figure S2: Effects of the EcN-pAIDA1-SP immunization on body temperature and gastrointestinal function.
