# Supplementary material for: Oral Immunization with Escherichia coli Nissle 1917 Expressing SARS-CoV-2 Spike Protein Induces Mucosal and Systemic Antibody Responses in Mice
Source: Biomolecules. 2023 Mar 21;13(3):569. doi: 10.3390/biom13030569 (PMC10046078; doi:10.3390/biom13030569)

Supplementary Material

1. Supplementary Methods.

1.1 Short-term immunization protocol.

In a preliminary set of experiments, we performed a short-term immunization protocol involving n=16 eight-week-old male C57BL/6 mice (Charles River, Lecco, Italy). The animals have been divided into 4 groups (n=4 each) and received either a single gavage of EcN-pAIDA1-SP 2 x 10^9 CFU (group 1) or repeated gavages of EcN-pAIDA1-SP 2 x 10^9 CFU for two, three or four consecutive days (groups 2, 3 and 4; respectively). Blood samples (collected from the tail vein) and stool samples were collected at days 7, 14, and 28 a processed as abovementioned for the detection of anti-SARS-CoV-2 Spike IgA and IgG (see Supplementary scheme 1

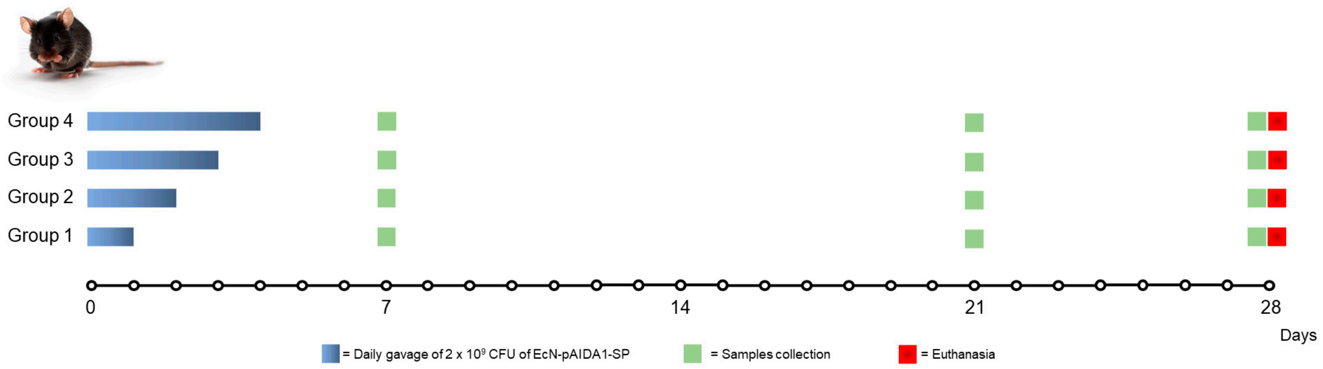

Supplementary scheme 1. Experimental time course for the short-term immunization.

1.2 Assessment of safety profile and adverse effects.

The Disease Activity Index (DAI) score was used to exclude gastrointestinal side effects. DAI index was calculated as total score (body weight decrease + stool consistency + rectal bleeding) divided by 3, as previously reported by Cooper et al. [23]. Bodyweight, stool consistency, and the presence of gross blood in stools were evaluated daily and scored for each mouse during the experimental period. Rectal temperature was measured daily for the entire duration of the experimental course. To obtain rectal temperature, the mice were hand-restrained and placed on a horizontal surface. The tail was then lifted, and the probe (covered with Vaseline) was gently inserted into the rectum to a fixed depth [24].

2. Supplementary Results.

2.1 Short-term immunization displayed ineffectiveness in stimulating Ig(s)-mediated response

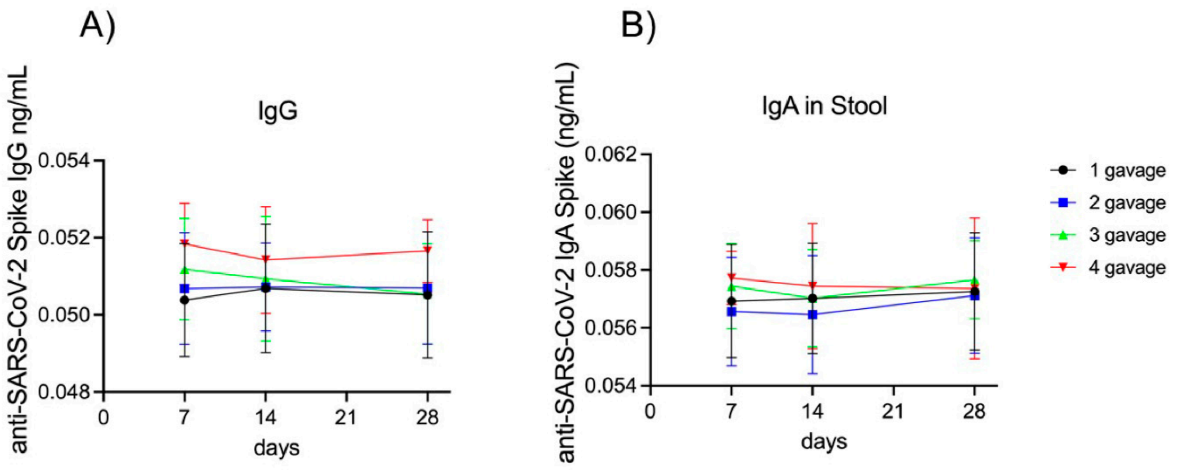

Supplementary Figure S1. ***Anti-SARS-CoV2 spike protein antibody titre (ng/mL) in serum IgG (A) and stool IgA (B) following a short-term immunization protocol with daily intragastric gavage at day 1, 2, 3, and 4 of EcN-pAIDA1-SP***. None of the animals displayed an increase in mucosal IgA nor in systemic IgG levels. The titre of Ig(s) remains flat in all of the experimental groups at all of the time points (days 7, 14, and 28 all p=NS). Results are expressed in mean ± SD of n=4 experiments performed in triplicate

2. EcN-pAIDA1-SP immunization did not induce side effects or affect mice survival.

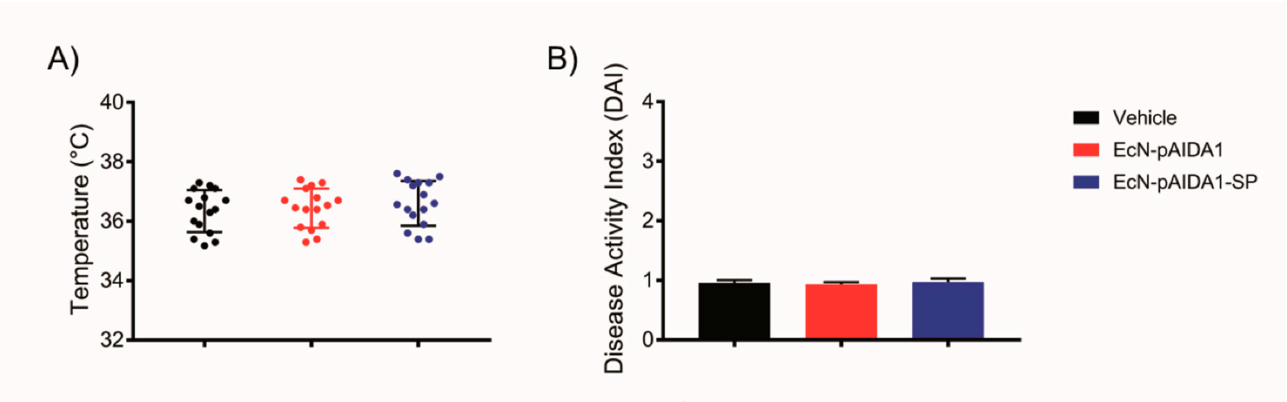

Supplementary Figure S2. ***Effects of EcN-pAIDA1-SP immunization on body temperature and gastrointestinal function.*** No significant differences were recorded in body temperature (daily measured) (A) and DAI (daily measured) (B) as compared to vehicle and control groups in EcN-pAIDA1-SP treated mice (all p= NS).

2.1 Uncropped blots from Figure 1.

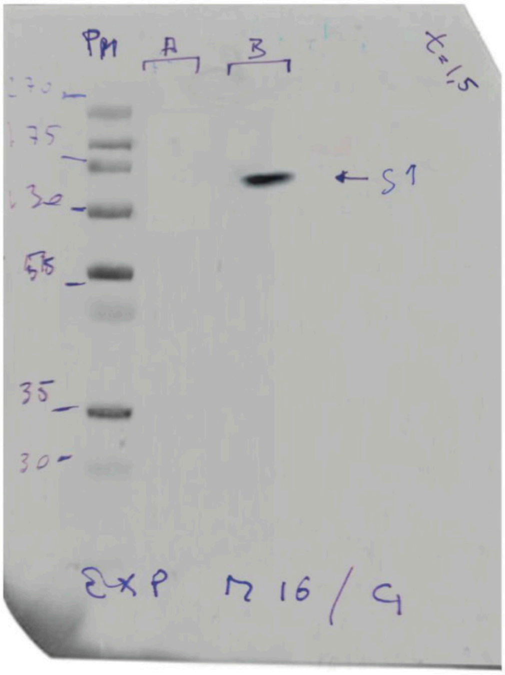

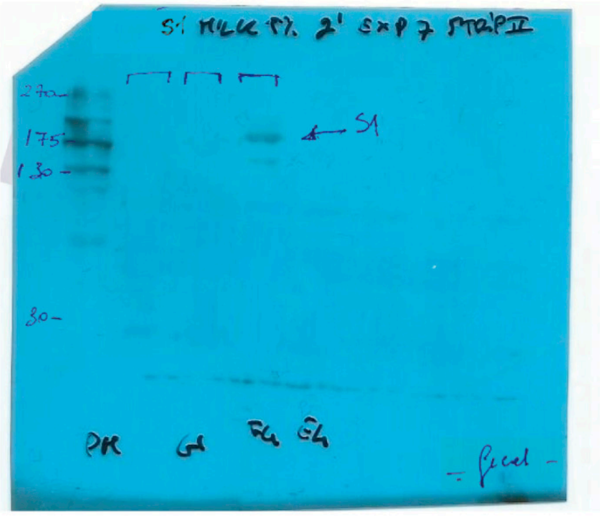

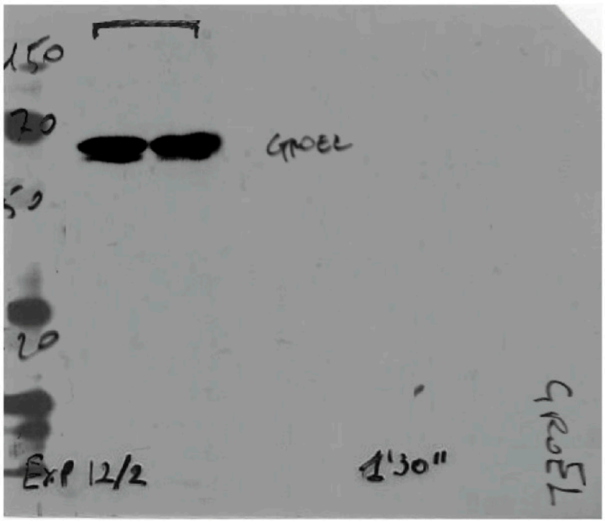

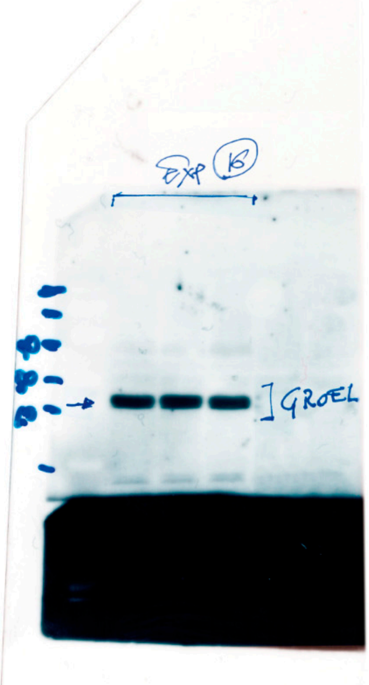

Supplement: Supplementary file 1 [file biomolecules-13-00569-s001.zip › biomolecules-2192672-supplementary.pdf]
